# Pronounced Metabolic Changes in Adaptation to Biofilm Growth by *Streptococcus pneumoniae*

**DOI:** 10.1371/journal.pone.0107015

**Published:** 2014-09-04

**Authors:** Raymond N. Allan, Paul Skipp, Johanna Jefferies, Stuart C. Clarke, Saul N. Faust, Luanne Hall-Stoodley, Jeremy Webb

**Affiliations:** 1 Academic Unit of Clinical and Experimental Sciences, Faculty of Medicine and Institute for Life Sciences, University of Southampton, Southampton, United Kingdom; 2 Southampton NIHR Wellcome Trust Clinical Research Facility, University Hospital Southampton NHS Foundation Trust, Southampton, United Kingdom; 3 Centre for Biological Sciences, University of Southampton, Southampton, United Kingdom; 4 Centre for Proteomic Research, Institute for Life Sciences, University of Southampton, Southampton, United Kingdom; 5 Public Health England, Southampton, United Kingdom; 6 Southampton NIHR Respiratory Biomedical Research Unit, University Hospital Southampton NHS Foundation Trust, Southampton, United Kingdom; 7 Microbial Infection and Immunity, Centre for Microbial Interface Biology, The Ohio State University, Columbus, Ohio, United States of America; University of Oklahoma Health Sciences Center, United States of America

## Abstract

*Streptococcus pneumoniae* accounts for a significant global burden of morbidity and mortality and biofilm development is increasingly recognised as important for colonization and infection. Analysis of protein expression patterns during biofilm development may therefore provide valuable insights to the understanding of pneumococcal persistence strategies and to improve vaccines. iTRAQ (isobaric tagging for relative and absolute quantification), a high-throughput gel-free proteomic approach which allows high resolution quantitative comparisons of protein profiles between multiple phenotypes, was used to interrogate planktonic and biofilm growth in a clinical serotype 14 strain. Comparative analyses of protein expression between log-phase planktonic and 1-day and 7-day biofilm cultures representing nascent and late phase biofilm growth were carried out. Overall, 244 proteins were identified, of which >80% were differentially expressed during biofilm development. Quantitatively and qualitatively, metabolic regulation appeared to play a central role in the adaptation from the planktonic to biofilm phenotype. Pneumococci adapted to biofilm growth by decreasing enzymes involved in the glycolytic pathway, as well as proteins involved in translation, transcription, and virulence. In contrast, proteins with a role in pyruvate, carbohydrate, and arginine metabolism were significantly increased during biofilm development. Downregulation of glycolytic and translational proteins suggests that pneumococcus adopts a covert phenotype whilst adapting to an adherent lifestyle, while utilization of alternative metabolic pathways highlights the resourcefulness of pneumococcus to facilitate survival in diverse environmental conditions. These metabolic proteins, conserved across both the planktonic and biofilm phenotypes, may also represent target candidates for future vaccine development and treatment strategies. Data are available via ProteomeXchange with identifier PXD001182.

## Introduction

Pneumococcal disease accounts for a significant global burden of morbidity and mortality, particularly in younger children and older adults. *Streptococcus pneumoniae* is a Gram-positive facultative anaerobe that colonises the majority of children in the first year of life and is associated with mostly asymptomatic carriage in the human respiratory tract. However, multifactorial and incompletely understood mechanisms allow *S. pneumoniae* to cause localised infection such as otitis media and sinusitis, and/or invasive disease (meningitis, bacteremia and pneumonia). The transition from asymptomatic colonisation of the respiratory mucosal epithelium without inflammation to the initiation of infection is thought to correlate with the expression of specific cell surface molecular properties such as adhesins and capsule polysaccharides under different conditions [1]. Although pneumococcal conjugate vaccines have reduced carriage and disease caused by capsular strains included in these vaccines, there has been a rise in the prevalence of capsular strains not included in the vaccines (serotype replacement). With over 90 different pneumococcal serotypes, there is a need for more effective vaccines that are conserved across serotypes, immunogenic in all age groups, and effective against both colonisation and infection by *S. pneumoniae* [2].

Aggregated pneumococci adherent to human middle ear mucosae of children undergoing treatment for chronic otitis media (COM) indicate that *S. pneumoniae* is present in biofilms on the mucosal epithelium [3], an observation supported by animal models [4], [5]. Pneumococcal biofilms were also observed on adenoid mucosal epithelium from children undergoing adenoidectomy for the treatment of obstructive sleep apnea, suggesting that pneumococcal biofilms are present in the nasopharynx in both the presence and absence of overt infection [6], [7]. The ability of *S. pneumoniae* to develop biofilms has been shown to affect both colonisation and disease states [8]–[10], and understanding *S. pneumoniae* biofilm development is important for studying the mechanisms of persistence.

*S. pneumoniae* adapts to different environments in the human host by modulating proteins and carbohydrates that may function as virulence factors. In addition to different capsule serotypes, multiple proteins modulate cell attachment and contribute to both colonisation and invasion. *In vitro* studies with pneumococcal clinical strains have demonstrated that biofilm growth downregulates or disrupts capsule expression, and induces the production a complex extracellular matrix consisting of carbohydrates, proteins and DNA [11]–[14] and differential expression of a broad spectrum of proteins [15], [16]. We hypothesized that protein expression patterns during biofilm growth might highlight important phenotypic changes facilitating pneumococcal persistence or virulence. On the other hand, conserved protein expression in both planktonic and biofilm growth, might inform better vaccine strategies since these proteins would be present regardless of the mode of growth or type of infection.

Serotype 14 is a common serotype in pneumococcal infection worldwide, causing both localised and invasive disease. It exhibits high levels of antibiotic resistance [17] and is prevalent in paediatric disease such as otitis media [18]. Serotype 14 was chosen in this study as a model strain because of its high biofilm-forming index based on multiparametric analysis of different clinical *S. pneumoniae* strains [13]. Previous proteomic analyses of *S. pneumoniae* growth have used conventional 1DE and 2DE gel-based methodologies [15], [16], [19]–[21]. iTRAQ (isobaric tag for relative and absolute quantitation) is a high-throughput gel-free proteomic profiling approach enabling quantitative high resolution comparison of protein profiles between multiple phenotypes or growth conditions within a single experiment. To investigate the pneumococcal serotype 14 biofilm proteome, we compared nascent 1-day biofilms lacking complex structure, highly structured 7-day biofilms, and log-phase planktonic growth to determine: 1) distinct patterns of differential expression comparing planktonic, nascent and late-phase biofilm growth, and 2) conserved patterns of protein regulation during planktonic and biofilm growth.

## Methods

### Bacterial strain and growth conditions

A Serotype 14 (ST124) blood sample isolate provided by the UK Public Health England (Health Protection Agency) Microbiology Services laboratory, Southampton, was selected based on multi-parametric, statistically-based criteria for biofilm development [13] [personal observation RNA/LHS]. The strain was subcultured from a frozen stock onto Columbia blood agar (CBA) plates (Oxoid, U.K.) and incubated for 14 hours at 37°C/5% CO_2_. Colonies were re-suspended in 2 ml Brain Heart Infusion (BHI) broth (Oxoid, U.K.), centrifuged at 3,200×*g* for 5 min to remove cell debris and secreted lytic enzymes (personal communication, Public Health England), and the supernatant used to inoculate fresh dilute BHI broth. For planktonic growth, cultures were grown to mid-exponential phase (OD_600_ = 0.3; ∼1×10^8^ cells).

### Biofilm Formation

For biofilm formation, mid-exponential planktonic cultures (2 ml×10^8^ cells) were used to inoculate individual wells of polystyrene 6-well plates (Corning Incorporated, Costar, U.S.A.), and all wells supplemented with 2 ml BHI diluted 1∶5 with non-pyrogenic sterile dH_2_O. Cultures were incubated under static conditions at 37°C/5% CO_2_ with replacement of warm, fresh diluted BHI daily.

### Colony Forming Unit (CFU) Enumeration and Total Cell Counts

All medium was removed and biofilms washed twice using diluted BHI. Biofilms were resuspended in 1 ml Hank’s balanced salt solution (HBSS) as described [13]. Briefly, bacterial suspensions (n = 3) underwent 10-fold dilutions in HBSS and 5×20 µl of each dilution spot plated onto CBA plates and incubated at 37°C/5% CO_2_. For total cell counts (n = 3) bacterial suspensions were diluted 10-fold, stained with Syto 9 (Life Technologies, U.S.A.) according to manufacturer instructions, and individual cells counted using a haemocytometer (Marienfeld-Superior, Germany) and a Leica epifluorescent microscope using a 100x oil immersion lens.

### Scanning Electron Microscopy (SEM)

Biofilms (n = 3) were grown in 6-well plates containing ethanol-sterilized 13 mm glass coverslips (V.W.R., U.K.) under static conditions at 37°C/5% CO_2_. All medium was removed and biofilms washed twice using diluted BHI. Coverslips were removed using sterile forceps, placed in primary fixative solution (3% gluteraldehyde, 0.1 M sodium cacodylate (pH 7.2), 0.15% Alcian blue), and incubated at 4°C overnight. The primary fixative was replaced with 0.1 M sodium cacodylate (pH 7.2), followed by the secondary fixative solution (0.1 M osmium tetroxide, 0.1 M sodium cacodlyate; pH 7.2), and finally with 0.1 M sodium cacodylate (pH 7.2). Each treatment was incubated for 1 h at room temperature. Coverslips were processed through an ethanol series to 100%, critical point dried, and sputter coated and biofilms imaged using FEI Quanta 200 scanning electron microscope.

### Confocal Laser Scanning Microscopy (CLSM)

Biofilms (n = 6) were grown in 35 mm glass bottom microwell dishes (MatTek Corporation, U.S.A.) under static conditions at 37°C with 5% CO_2_, washed twice with HBSS and stained with LIVE/DEAD BacLight Bacterial Viability Kit (Life Technologies, U.S.A.) according to manufacturer instructions. Biofilms were examined immediately using a Leica SP5 LSCM with inverted stand using a 63x oil immersion lens, performing sequential scanning using 0.5 µm sections. Three random fields of view were captured and images were analysed using Leica LCS Software.

### Protein Extraction

For planktonic protein samples, cultures were grown to mid-exponential phase and centrifuged. The supernatant was discarded and the pellet washed twice in 1 ml Hanks’ Balanced Salt Solution (HBSS; Gibco, Life Technologies, U.K.) at 9,500×*g*. For biofilm protein samples 1 and 7 day cultured biofilms were washed twice with HBSS and resuspended in 1 ml HBSS using sterile cell scrapers. The bacterial suspension was centrifuged (9,500×*g*), the supernatant removed and the pellet resuspended in 0.1 M triethylammonium bicarbonate (TEAB) buffer (Sigma-Aldrich, U.K.) with 0.1% Rapigest SF surfactant (Waters, U.K.). Both the planktonic and biofilm samples were lysed in lysing matrix B (MP Bioscience, U.K.) using a Hybaid Ribolyser Homogenizer (Hybaid, U.K.) in six 30 second sessions with 30 second storage on ice between sessions. The lysates were centrifuged at 855×*g*/5 min and the supernatant retained (soluble protein fraction). Protein quantification of the soluble protein fraction was performed using Coomassie Protein Assay Reagent (Thermo Fisher Scientific, U.K.), measuring optical density (595 nm) and quantified against albumin standards (Thermo Fisher Scientific, U.K.).

### iTRAQ labelling

Comparative analyses of protein expression between planktonic log-phase culture and 1-day and 7-day biofilms were performed on 3 technical replicates of 3 biological replicates. iTRAQ labelling of samples was performed according to the manufacturer’s instructions using an iTRAQ Reagent-8 plex Multiplex Kit (AB Sciex U.K. Limited). Protein samples were normalized to ∼60 µg and 2 µl of the reducing agent and 50 mM (tris-(2-carboxyethyl) phosphine were added and mixed. Samples were incubated at 60°C for 1 h. Methyl methane-thiosulfonate in isopropanol (1 µl of 200 mM) was then added and incubated for a further 10 min at room temperature. Proteins were digested by adding 10 µl of 1 mg/ml trypsin in 80 mM CaCl_2_. Samples were incubated for 16 h at 37°C. Labelling of tryptic peptides with iTRAQ 8-plex tags was achieved by mixing the appropriate iTRAQ labeling reagent with the relevant sample, and incubation at room temperature for 2 h.

### Mass Spectrometry

Two dimensional separations were performed using a nanoAcquity 2D UPLC system (Waters). For the first dimension separation, 2.0 µl of the prepared protein lysates containing 3.2 µg of iTRAQ labeled peptides were injected onto a 5 µm Xbridge BEH130 C18, 300 µm ID×50 mm (Waters) column equilibrated in 20 mM ammonium formate, pH 10 (buffer A). The first dimension separation was achieved by increasing the concentration of acetonitrile (buffer B) in 7 steps consisting of 11, 14, 16, 20, 25, 50, 65%. At each step the programmed percentage composition was held for 1 min at a low rate of 1000 nl/min and the eluant diluted by buffer C (H_2_0+0.1% formic acid) from the second dimension pump at a flow rate of 20 µl/min, effectively diluting the ammonium formate and acetonitrile, allowing trapping of the eluting peptides onto a Symmetry C18, 180 µm×20 mm trapping cartridge (Waters). After 20 min washing of the trap column, peptides were separated using an in-line second dimension analytical separation performed on a 75 µm ID×200 mm, 1.7 µm BEH130 C18, column (Waters) using a linear gradient of 5 to 40% B (buffer A = water containing 0.1% formic acid, buffer B = 95% acetonitrile containing 0.1% formic acid) over 120 min with a wash to 85% B at a flow rate of 300 nl/min. All separations were automated and performed on-line to the mass spectrometer.

All data were acquired using a Q-tof Global Ultima (Waters Ltd, Manchester, UK) fitted with a nanoLockSpray source. A survey scan was acquired from *m/z* 350 to 1700 with the switching criteria for MS to MS/MS including ion intensity and charge state. The collision energy used to perform MS/MS was varied according to the mass and charge state of the eluting peptide. Peak lists were generated using ProteinLynx Global Server 2.4 (Waters Ltd, Manchester, UK). The following parameters were used for processing the MS/MS spectra; normal background subtraction with a 25% background threshold and medium de-isotoping with a threshold of 1%, no smoothing was performed.

### Peak list generation and database searching

Peak lists from the MS/MS analysis of were submitted to the MASCOT search engine version 2.2.1 (Matrix Science, London, UK) and the data searched against a protein translation of the CGSP14 NCBI genome (http://www.ncbi.nlm.nih.gov/Taxonomy/Browser/wwwtax.cgi?id=516950). The corresponding quantitative information using the iTRAQ reporter ions was also obtained via MASCOT.

A maximum of one missed cleavage for tryptic digestion and fixed modifications for methyl methane-thiosulfonation of cysteine and the N-terminus and lysine side chains using the 8-plex iTRAQ label were allowed (Applied Biosystems, Warrington, UK). Variable modification for the oxidation of methionine and iTRAQ modification of tyrosine were also allowed. Precursor ion and sequence ion mass tolerances were set at 100 ppm and 0.3 Da respectively. Protein identifications required the assignment of ≥2 different peptides with a significance threshold for accepting a match of p<0.05. The protein ratios were calculated using MASCOT version 2.2.1, where the peptide ratios were weighted and median normalisation was performed, automatic outlier removal was chosen and the peptide threshold was set to ‘at least homology’. False discovery rates were calculated by running all spectra against a decoy database using the MASCOT software.

Inclusion criteria for quantitative analysis were set at ≥3 peptide matches, ≥50 protein score, ≥5% sequence coverage (p<0.05). Comparative protein data with >1.3 and <0.77 ratios were identified as having differential expression. For qualitative identification the inclusion criteria were ≥2 peptide matches, 50 protein score and 10% sequence coverage.

## Results

### Pneumococcus grew over 7 days from unstructured cell clusters to a structurally complex biofilm comprised of metabolically active, viable cells

**Figure 1:**
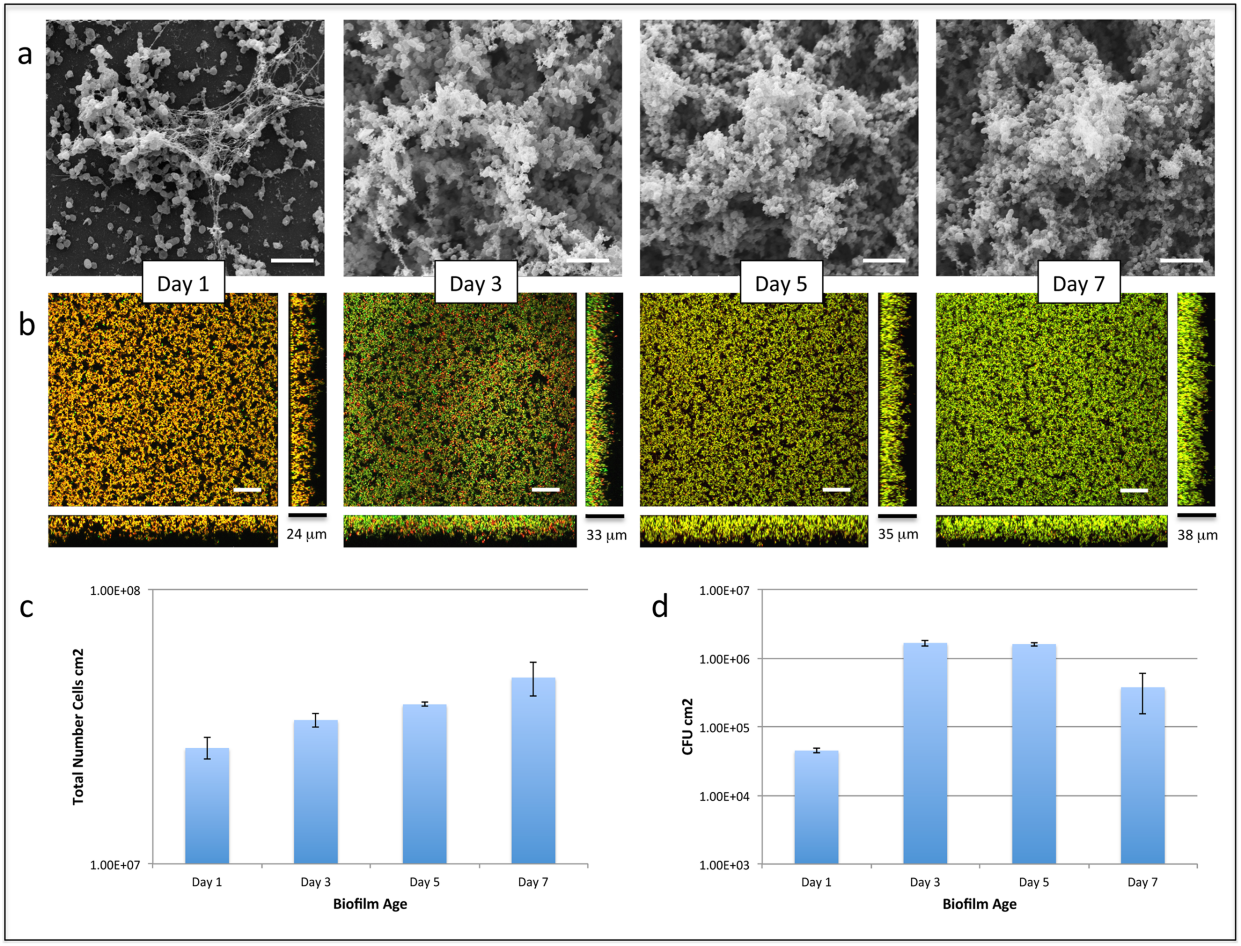
*S. pneumoniae* serotype 14 day 1, 3, 5 and 7 biofilms imaged using (a) scanning electron microscopy with Alcian Blue staining (8,000x magnification; scale bar: 5 µm), and (b) confocal microscopy using Live/Dead staining, with sideviews (YZ – right) and (XZ – bottom) sagittal sections of the biofilm. Scale bar beside YZ sagittal sections represents average biofilm thickness. Scale bar in xy pane: 30 µm. (c) Bar chart comparing the total number of cells between day 1, 3, 5 and 7 biofilms. (d) Bar chart comparing number of viable cells between day 1, 3, 5 and 7 biofilms through CFU cm^−2^ measurement.

SEM demonstrated that day 1 biofilms were characterized by the presence of small clusters of cells interspersed amongst individual cells and diplococci (Fig. 1a) surrounded by extracellular material linking numerous clusters together. Adherent growth after 7 days resulted in structurally complex biofilms characterized by large towers of cells connected by chains of cells. CLSM following Live/Dead staining demonstrated few live cells in day 1 biofilms, whereas day 7 biofilms were comprised of live cells adjacent to copious amounts of extracellular matrix (Fig. 1b). Day 7 biofilms also demonstrated increased numbers of metabolically active cells following Calcein violet staining (data not shown), and a ten-fold increase in CFU/cm^2^ compared with nascent (1-day) biofilms (Fig. 1c & 1d). These results agree with other reports showing that Live/Dead correlates with viability in pneumococcal biofilms [12]–[14].

### Pneumococcus exhibited extensive proteomic modulation during biofilm development

**Figure 2:**
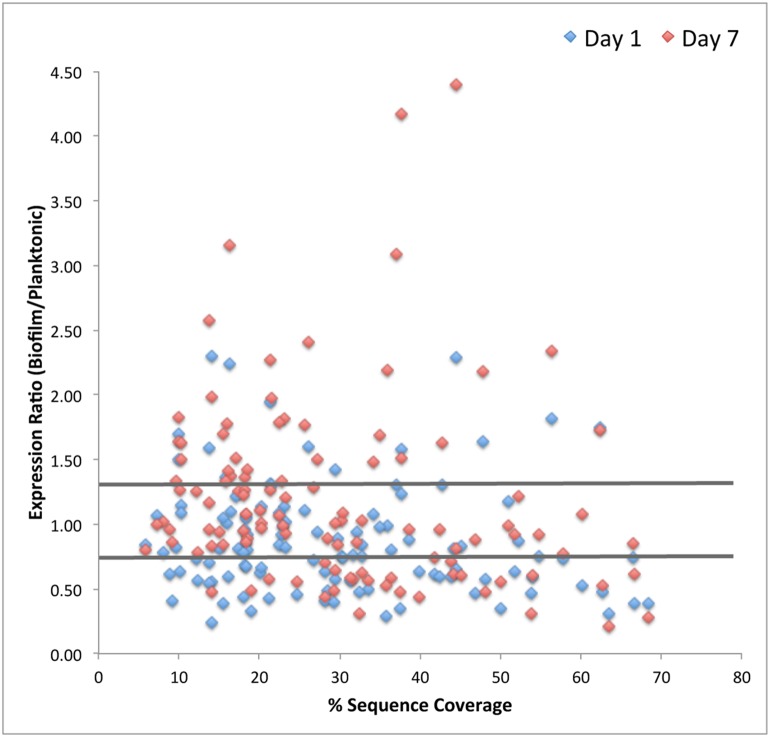
Volcano plot of the complete iTRAQ proteomic dataset comparing the fold change in protein expression between mid-exponential planktonic *S. pneumoniae* and day 1 and day 7 biofilms. Proteins represented met inclusion criteria of ≥3 peptide matches, ≥50 protein score, and ≥5% sequence coverage (p<0.05). Comparative protein data with >1.3 and <0.77 ratios (marked by horizontal lines) were identified as having differential expression.

**Figure 3:**
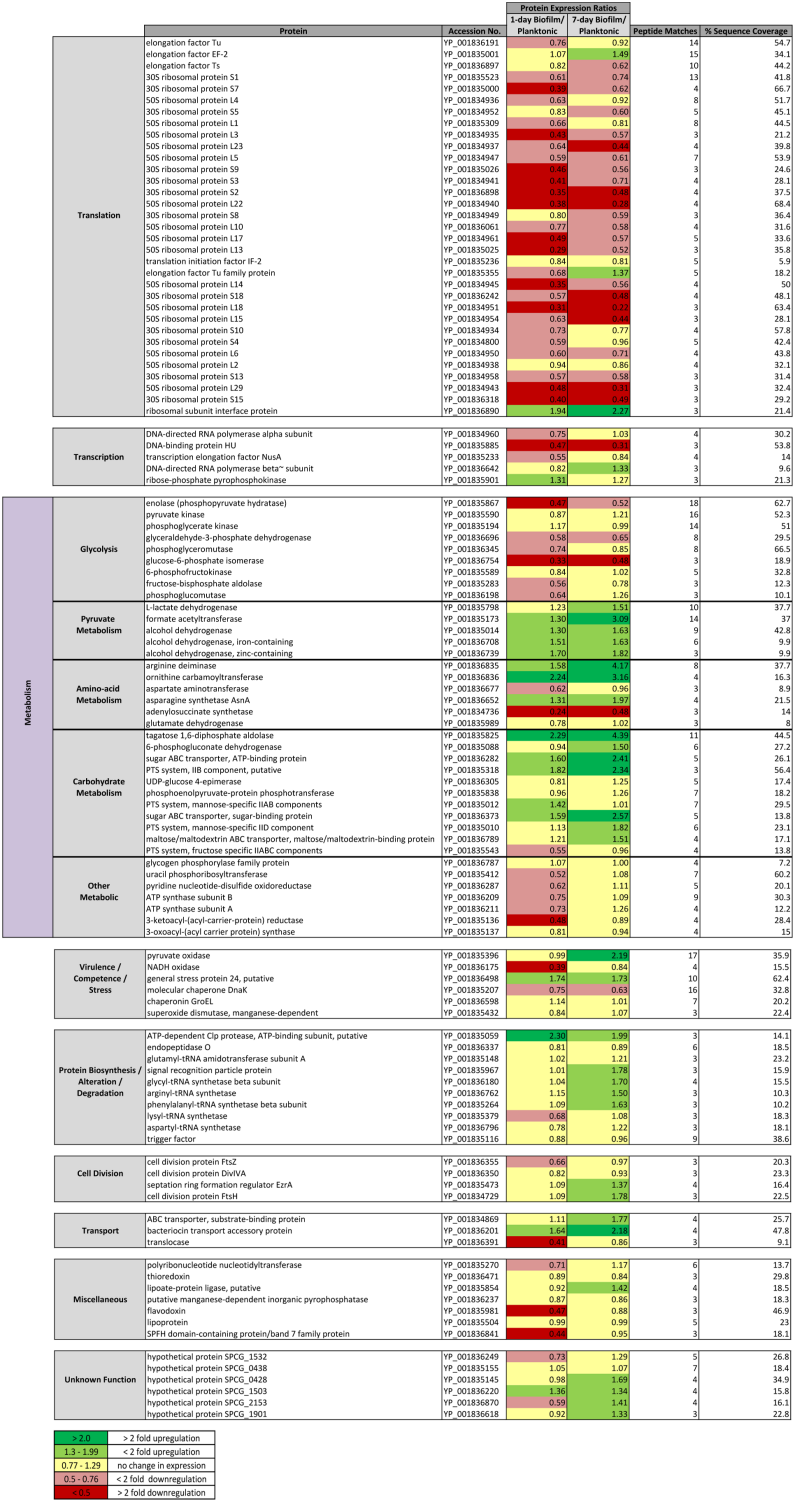
Comparative iTRAQ analyses of *S. pneumoniae* serotype 14 1-day and 7-day old *in vitro* biofilms to mid-exponential planktonic population protein expression. Inclusion criteria: ≥3 peptide matches, ≥50 protein score, ≥5% sequence coverage (p<0.05). Comparative protein data with >1.3 and <0.77 ratios identified as having differential expression.

**Figure 4:**
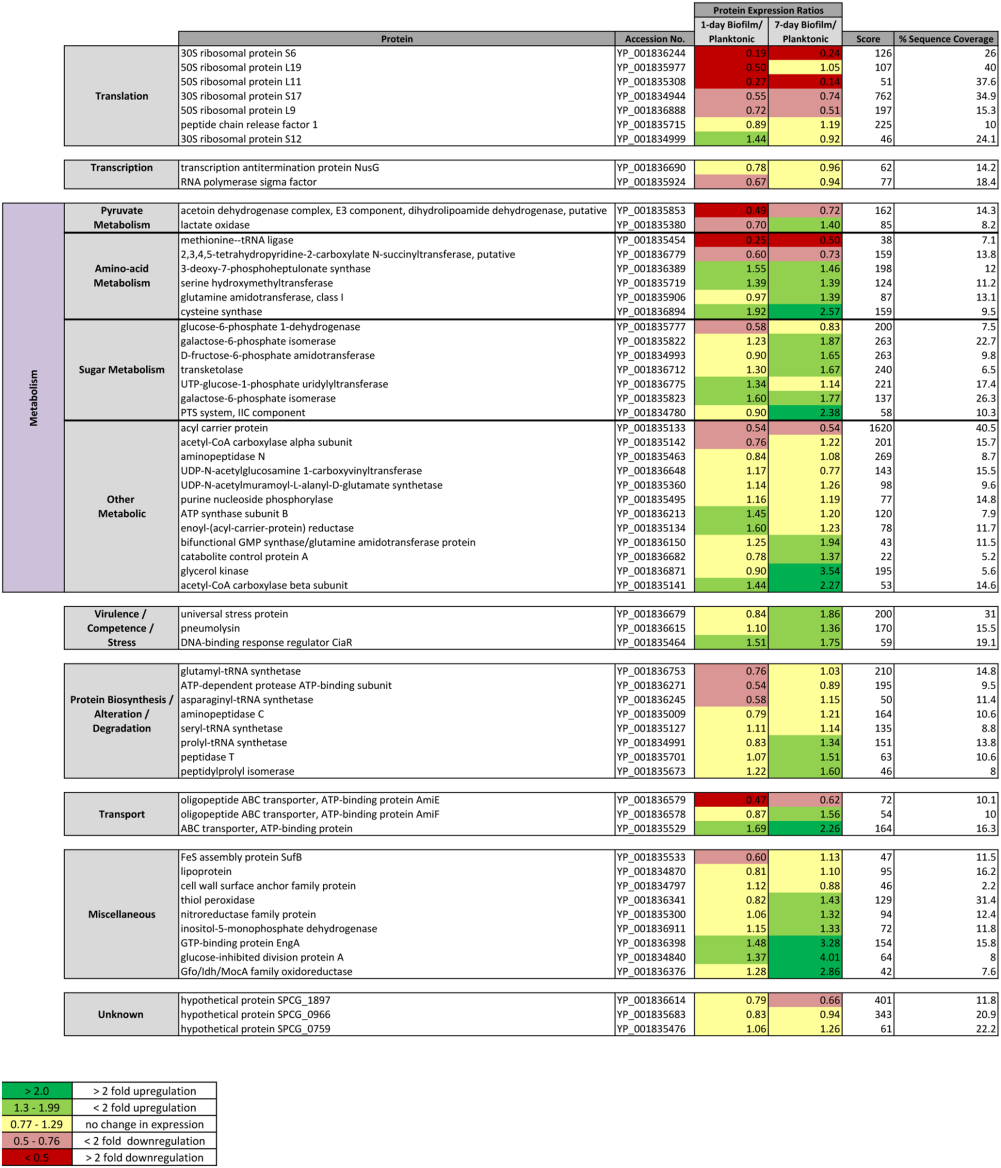
Qualitative data listing proteins identified with 2 peptide matches using comparative iTRAQ analysis of *S. pneumoniae* serotype 14 1-day and 7-day old *in vitro* biofilms to planktonic population protein expression. Comparative protein data with >1.3 and <0.77 ratios identified as having differential expression.

Overall, 244 individual proteins were identified using iTRAQ profiling of which 112 met inclusion criteria for quantitative analysis (≥3 peptide matches; >5% sequence coverage and a 50+ score; P<0.05) (Fig. 2 & 3). Additionally, 62 proteins with 2 peptide matches were qualitatively identified (Fig. 4). The majority (>80%) of proteins were differentially expressed during biofilm development. Nascent (1-day) pneumococcal biofilms differed significantly from log-phase planktonic culture with 47% (53/112) of identified proteins down-regulated and only 16% (18/112) up-regulated at this time point (Fig. 2).

Adherent growth for 7 days resulted in further changes in protein expression compared with early biofilm growth with 44% of proteins (49/112) overall differentially expressed. Nearly a quarter (24%; 27/112) of the proteins differentially expressed in the nascent 1-day old biofilm returned to levels similar to log-phase planktonic growth in the late-phase 7-day old biofilm, and a further 16% (18/112) proteins were up-regulated; only 3/112 (2.7%) were down-regulated in late compared with early biofilm growth. When late biofilm growth was compared with log-phase planktonic protein expression 35/112 proteins (31%) were up-regulated, 48/112 (43%) were similarly expressed, and 29/112 (26%) were down-regulated. Nearly 20% (21/112) of proteins were quantitatively unchanged in all profiles. iTRAQ profiling suggests the transition to adherent growth was a dynamic adaptive process consistent with previous proteomic analyses demonstrating remarkable changes in expression profiles during pneumococcal biofilm development. Pneumococcal translational capacity was substantially reduced during biofilm development. Of the 33 identified proteins specific to translation, 29 (88%) were down-regulated with over half (15/29) more than two-fold, together with increased levels of a ribosomal subunit interface protein, which facilitates arrest of translation [22].

### Glycolytic proteins were significantly decreased during nascent pneumococcal biofilm development

Previous proteomic analyses of *S. pneumoniae* indicate that biofilm and planktonic pneumococci substantially differ in a number of proteins involved in biosynthesis and metabolism [15], [16], [21], and iTRAQ profiling broadly supported these. Thirty-eight metabolic proteins were quantitatively identified and divided into 4 subsets specific to glycolysis, amino acid, carbohydrate and pyruvate metabolism, with a final group comprised of proteins with miscellaneous metabolic roles (Fig. 3). Of all metabolic proteins quantified, 37% (14/38) were decreased in the nascent biofilm, and 32% (12/38) increased. After 7 days of adherent growth however, all but 4 (11%) proteins initially down-regulated in the nascent biofilm, returned to levels similar to log-phase planktonic growth. Additionally, 40% (15/38) of metabolic proteins were increased in late biofilm growth suggesting that pneumococcal biofilms adapted to adherent growth, exhibiting protein levels observed in log-phase growth or up-regulating specific proteins associated with pyruvate, sugar and amino acid metabolism (Fig. 3).

**Figure 5:**
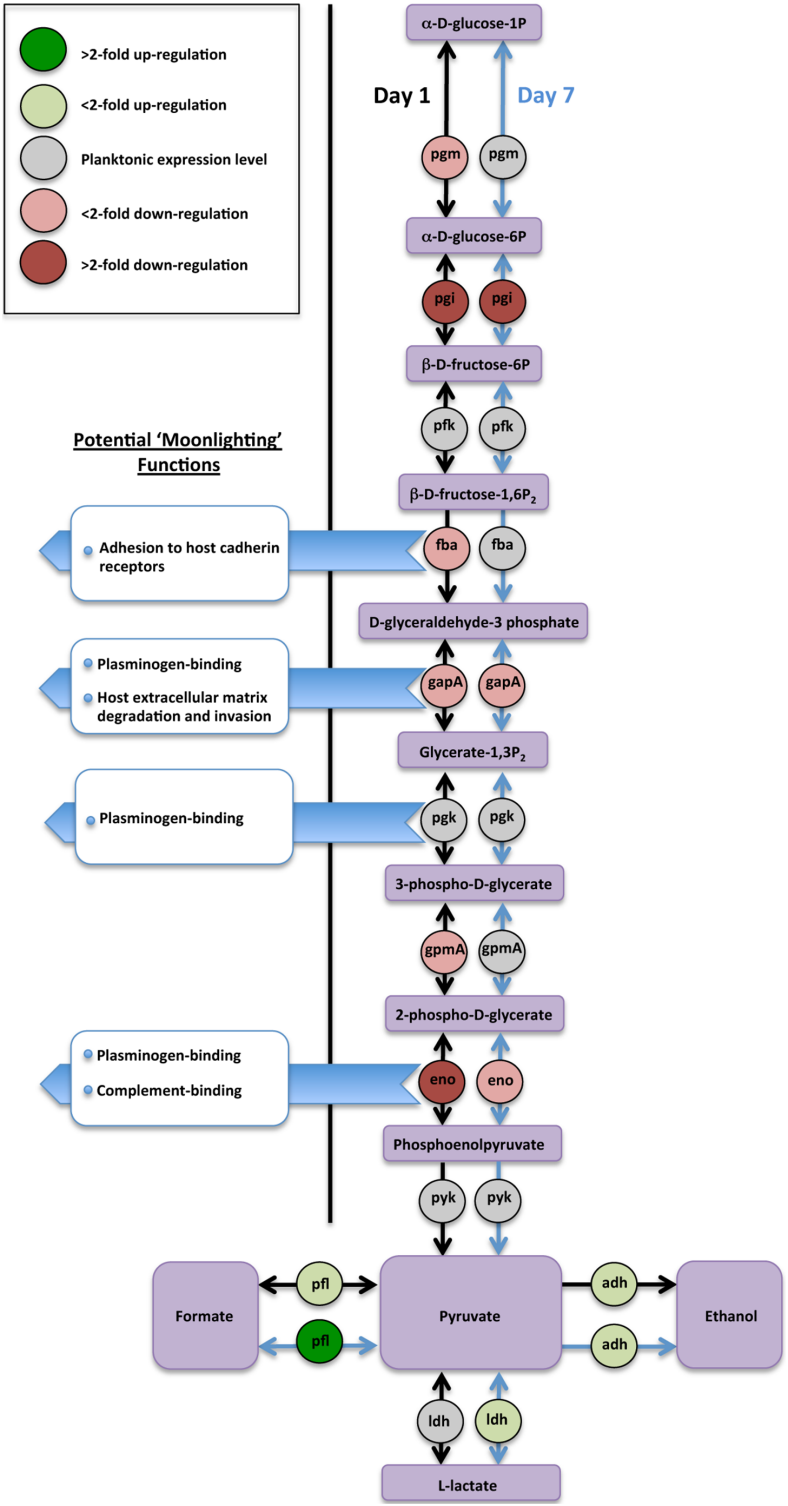
Change in expression of enzymes involved in the glycolysis/gluconeogenesis pathway and pyruvate metabolism of *S. pneumoniae* serotype 14 during biofilm formation. Based on iTRAQ expression data comparing mid-exponential planktonic cultures to day 1 and 7 biofilms. Proteins with potential moonlighting capability highlighted with their putative alternative functions. Pgm - phosphoglucomutase; pgi – glucose-6-phosphate isomerase; pfk–6-phosphofructokinase; fba – fructose biphosphate aldolase; gapA – glyceraldehyde-3-phosphate dehydrogenase; pgk – phosphoglycerate kinase; gpmA – phosphoglyceromutase; eno – enolase; pyk – pyruvate kinase, ldh – lactate dehydrogenase; adh – alcohol dehydrogenase, pfl – formate acetyltransferase.

Compared with previous pneumococcal proteomic analyses, iTRAQ identified all 9 proteins mediating the glycolytic (Embden-Meyerhof-Parnas) pathway allowing us, for the first time, to fully and quantitatively characterize changes in the pathway during biofilm formation. The majority of these proteins were decreased in nascent biofilm development consistent with other reports demonstrating down-regulation of glycolytic proteins in early TIGR4 biofilms and following epithelial cell contact [23], [24]. However, after 7 days of adherent growth, half of these proteins were quantitatively commensurate with levels observed in log-phase planktonic growth (Fig. 3 & 5). All proteins associated with pyruvate metabolism were up-regulated in adherent pneumococci (Fig. 3 & 5).

### Proteins involved with amino-acid metabolism and carbohydrate utilization were up-regulated in adherent pneumococci

**Figure 6:**
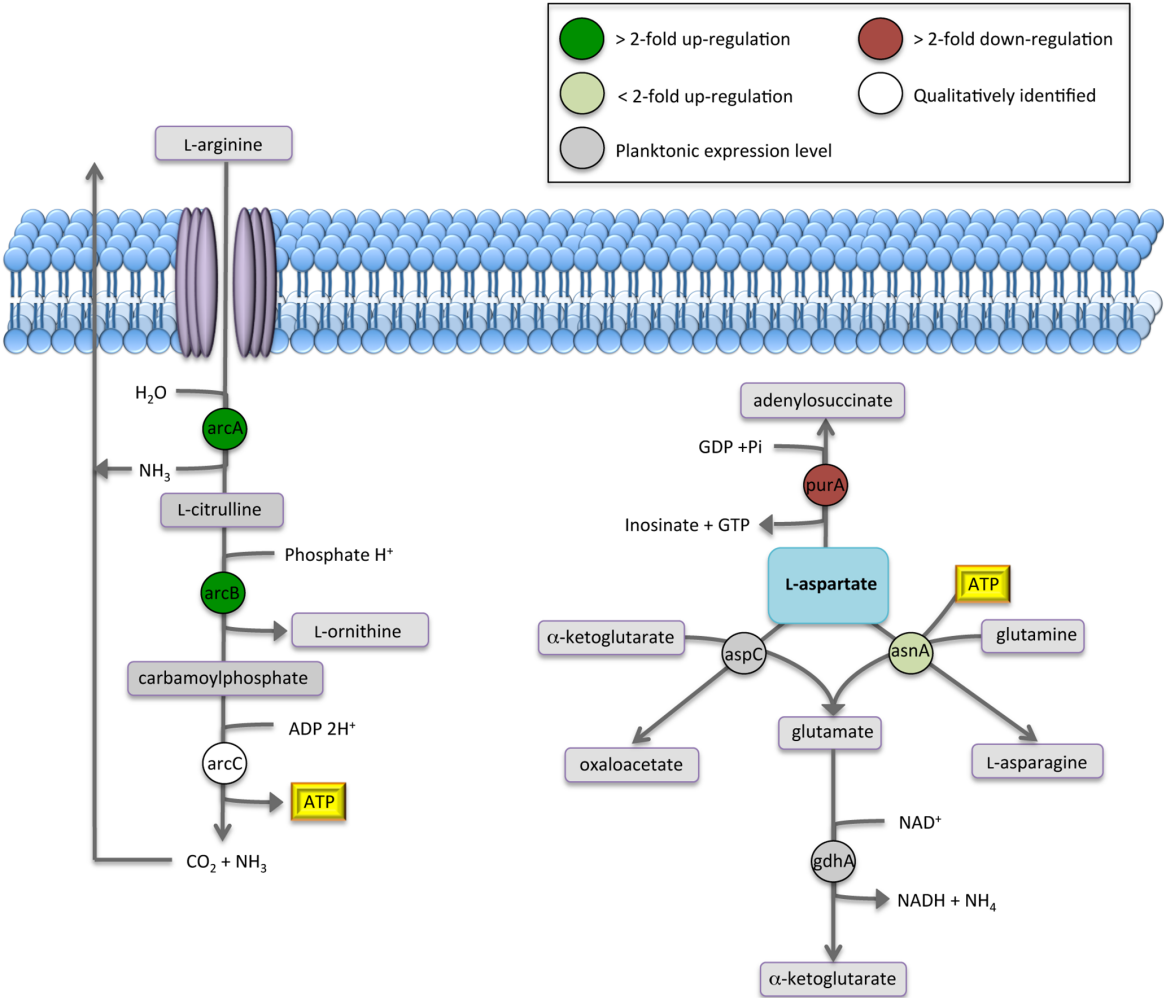
Change in expression of enzymes involved in amino-acid metabolism of day 7 *S. pneumoniae* serotype 14 biofilms. Based on iTRAQ expression data comparing mid-exponential planktonic cultures to day 7 biofilms. Highlighting the arginine deiminase (ADI) pathway consisting of arginine deiminase (arcA), ornithine carbamoyltransferase (arcB) and carbamate kinase (arcC), and components of L-aspartate metabolism consisting of adenylosuccinate synthetase (purA), aspartate aminotransferase (aspC), asparagine synthetase (asnA), and glutamate dehydrogenase (gdhA).

In contrast to the decrease in glycolytic proteins in adherent bacteria, there was a significant increase in proteins involved in amino acid and sugar metabolism. Analysis of these proteins revealed that 47% (8/17) were increased in nascent biofilms, and 3/17 (18%) were decreased. By day 7 59% (10/17) were significantly increased, and only one decreased, suggesting that adherent pneumococci utilized alternative metabolic pathways during biofilm development. Six proteins associated with amino acid metabolism were identified, 3 of which were up-regulated in both nascent and 7 day biofilms (Fig. 3 & 6). Arginine deiminase (ADI) and ornithine carbamoyltransferase, enzymes which facilitate the degradation of L-arginine to generate 1 mol ATP, were up-regulated >3-fold (Fig. 3 & 6). Aspartate aminotransferase and adenyolsuccinate synthetase were down-regulated in adherent pneumococci, while glutamate dehydrogenase corresponded with planktonic levels (Fig. 3 & 6).

Our data suggest *S. pneumoniae* utilised a range of carbohydrates during biofilm development, since 90% (10/11) of the proteins associated with sugar metabolism were either up-regulated or unchanged during nascent biofilm growth relative to log-phase planktonic growth. Furthermore, (64%; 7/11) proteins were significantly increased in day 7 biofilms with the remainder consistent with planktonic *S. pneumoniae* (Fig. 3). Five PTS system proteins were identified, with mannose-specific IIAB and IID components, and a putative IIB galactitol-specific component up-regulated. The phosphoenolpyruvate-protein (PEP) PTS, an active-transport system specific for carbohydrates, was unchanged between log-phase planktonic and adherent bacteria. A fructose II ABC component, was down-regulated in nascent biofilms, but commensurate with planktonic growth at day 7 biofilms (Fig. 3).

Additionally, three ABC transporter system proteins were significantly up-regulated in biofilm pneumococci (Fig. 3). An ATP-binding protein and a sugar-binding protein from a sugar ABC transporter were both increased >2 fold in day 7 adherent *S. pneumoniae* and correspond to *msmK* and the sialic acid ABC transporter protein (*SatABC*). A multi-substrate ABC transporter (maltose/maltodextrin-binding protein of the maltose/maltodextrin ABC transporter) corresponding to *MalX* was also up-regulated in the pneumococcal biofilm profile. Tagatose 1,6-diphosphate aldolase demonstrated the highest levels of expression in the biofilm phenotype (>4-fold at day 7). Several additional sugar metabolism proteins were identified qualitatively (Fig. 4), including a PTS system IIC component and another ABC transporter.

### Proteins associated with pneumococcal virulence and stress response were quantitatively different in biofilms

There was a >2-fold decrease in the level of NADH oxidase (nox) in nascent *S. pneumoniae* biofilms similar to a previous report [9], however, in day 7 biofilms, levels were similar to planktonic levels (Fig. 3). Superoxide dismutase (SOD) was unchanged between log-phase planktonic and biofilm pneumococci, indicating a requirement for protection from molecular oxygen in both conditions. Pyruvate oxidase, required for most stages of infection was also quantitatively unchanged between nascent and planktonic culture, but was >2-fold higher during day 7 biofilms. Bacterocin transport accessory protein (Bta) was also significantly higher (>2-fold) at this timepoint, suggesting increased production of bacteriocins by biofilm pneumococci. Stress proteins GroEL and DnaK, chaperone proteins facilitating appropriate protein conformation, and trigger factor were either decreased or unchanged compared with planktonic bacteria, however the pneumococcal general stress protein 24 (gls24) was increased in both nascent and late phase biofilms.

## Discussion

Biofilm growth by S. *pneumoniae* is incompletely understood, but mounting evidence suggests that it plays a role in colonisation and the ability to persist in the host [3], [4], [7]–[16], [23], [24]. We used a static biofilm system to model conditions present in chronic otitis media where there is an absence of flow [3], [13] to interrogate the hard-wired, intrinsic ability of *S. pneumoniae* to develop biofilms and obtain sufficient quantities of protein for analysis, without the presence of proteins from cell lines or animal cells to interfere with quantitative proteomic analysis.

Our data (to our knowledge the first study of *S. pneumoniae* using a quantitative gel-free approach and the first proteomic analysis of biofilms using a serotype 14 clinical strain) identify proteins whose role in biofilm development has previously been uncharacterized. iTRAQ analyses suggest that the regulation of metabolism plays a pivotal role during pneumococcal adaptation to aggregated biofilm growth. The glycolytic pathway, for which a quantitative profile of all enzyme components has been characterized for the first time, was generally down-regulated. In contrast, there was significant up-regulation of proteins involved in carbohydrate, pyruvate and arginine metabolism. These findings suggest that targeting proteins essential for pneumococcal adaption to biofilm growth, and particularly those involved in sugar and arginine metabolism, may offer new targets for treating pneumococcal-associated infections. Several proteins were conserved in both the planktonic and biofilm phenotypes, and may represent candidates for protein-based vaccines.

Protein profiling highlighted pronounced changes in the pneumococcal proteome during adaptation to the biofilm phenotype consistent with previous studies [15], [24]. Proteins related to translation, transcription, virulence and glycolysis were significantly decreased, particularly in nascent biofilms. Together with experiments showing many dead and metabolically inactive cells in nascent biofilms, pneumococcus appeared to adopt a quiescent phase while it adapted to biofilm growth. These results also suggest this time point may be characterized by increased pneumococcal lysis with lysed bacteria contributing substantially to the extracellular matrix, since *LytA* mutants failed to produce biofilms with an extracellular matrix [14]. These results are also similar to reports showing that pneumococcal biofilms are suggestive of a quiescent phase that may not be strongly immunogenic [16], [23]. After 7 days however, 2/3 of identified proteins quantitatively increased, with most of the remaining proteins statistically similar to log-phase planktonic growth. These data correlated with increased CFU/cm^2^, and viable, metabolically-active biofilm pneumococci *in situ* suggesting that biofilms were comprised of both viable and dead cells during adaption to biofilm growth (Fig. 1).

This nascent phase may be similar to the prioritization of carbon source utilization recently observed in planktonic lag phase growth in *E. coli* [25]. Moreover, the decrease in proteins associated with cell division and translation may be important in the decreased susceptibility of biofilm pneumococci to antibiotics that target the replication machinery and protein and cell wall synthesis [13], [14], [23], [26]. Even a small population of metabolically active cells in structurally complex biofilms would comprise a reservoir of viable pneumococci with the potential for propagation to new sites within the host [1]. Notably, other reports indicate that biofilm *S. pneumoniae* are hyperadhesive [23], [27]. However, biofilm development is multifactorial with disease potential related to clinical isolate, extracellular matrix production and previous cultivation with epithelial cells [12]–[14], [28], [29].

In our in vitro study, a key difference in planktonic and biofilm proteomic profiles was the expression of metabolic proteins. This is the first study to provide quantitative analysis of the complete set of glycolytic enzymes and indicated reduced levels of the majority of these proteins during the adaptation to biofilm growth, consistent with previous data showing decreased expression of 6 glycolytic proteins [16]. However, our analysis at a later time point in biofilm development suggests that further biofilm development resulted in half of these proteins returning to levels similar to planktonic pneumococci by day 7. Interestingly, 4 glycolytic proteins represent potential “moonlighting” proteins comprising a class of anchorless cell membrane proteins with dual protein binding and metabolic functions [30], [31]. Phoshopyruvate hydratase (α-enolase), glyceraldehyde-3-phosphate dehydrogenase (GapDH) and fructose bisphosphate aldolase (Fba) were significantly decreased in pneumococcal biofilms, whereas phosphoglycerate kinase (Pgk) was unchanged. Decreased expression of these proteins in *in vitro* biofilms suggests that *S. pneumoniae* modulated these bifunctional proteins upon adapting to biofilm growth and may promote a non-invasive phenotype capable of reducing an inflammatory response that facilitates persistence [1]. Given the streamlined pneumococcal genome, the ability of proteins to perform different functions depending on their localization in the cell would be a resourceful adaptation. Consistent with this possibility an SPFH domain-containing protein/band 7 family protein was decreased in nascent biofilms, but increased >2-fold in day 7 biofilms. These lipid raft-associated proteins aggregate to form membrane micro-domains that function in the recruitment of multi-protein complexes.

Evidence of increased pyruvate metabolism, and particularly lactate dehydrogenase, suggest that pneumococcal biofilm cells respond to a changing oxygen environment over time. The marked increase in arginine deiminase and ornithine carbamoyltransferase, two of three enzymes comprising the ADI system in pneumococcal biofilms, warrant further study. The ADI system has been shown to play a role in pathogenesis, energy metabolism and the protection of streptococcal biofilms from acidic conditions [32], and is widely distributed among bacteria with conservation of structure, but diversification of operons and regulation [33]. In particular, ADI has been characterized in oral streptococci where it plays a role in protection from lethal acidic conditions, production of ATP and survival. Recently, extracellular *Streptococcus intermedius* ADI was shown to inhibit *Porphyromonas gingivalis* biofilm formation by an interspecies signaling mechanism [33]. ADI in other streptococci has been shown to inhibit PBMC proliferation to be up-regulated in response to human serum [34], [35].

iTRAQ quantitative data indicate that carbohydrate metabolism is especially important during pneumococcal adaptation to biofilm growth. Proteins associated with carbohydrate utilization, including phosphotransferase system (PTS) and ABC transporter proteins that determine carbohydrate substrate selection and fermentation were significantly increased in biofilm bacteria [36]–[39] suggesting that *S. pneumoniae* was capable of metabolising a wide range of carbohydrates during biofilm development. Several PTS systems and ABC transporters, many of which have been shown to play a role in pathogenesis [36], were upregulated in the biofilm including a mannose-specific IIAB component up-regulated in 1 day adherent bacteria and mannose-specific IID significantly up-regulated at 7-days (Fig. 3). These membrane spanning proteins, corresponding to the ManL and ManN nomenclature in D39 and TIGR 4, are widely conserved and have been shown to transport glucose and galactose, GlcNAc and GlcN, in addition to mannose [37], [40]. The phosphoenolpyruvate-protein (PEP) PTS, an active-transport system specific for carbohydrates phosphorylating incoming sugar substrates during translocation across the cell membrane, was unchanged between planktonic and biofilm pneumococci suggesting this system was active in both growth conditions consistent with PEP’s role in pyruvate metabolism. Inhibition of PTS systems demonstrated attenuated virulence in *Staphylococcus aureus* and *Haemophilus influenzae* [41].

Several ABC transporter system proteins were significantly up-regulated in pneumococcal biofilms. A multi-substrate ABC transporter (maltose/maltodextrin-binding protein of the maltose/maltodextrin ABC transporter) corresponding to *MalX* was identified along with several additional sugar metabolism proteins. Pneumococcal ABC transporters may utilise multiple carbohydrates as nutrient substrates [36], [37], [40], since free carbohydrates are scarce in the human nasopharynx whereas complex glycans are abundant. Specifically, an ATP-binding protein corresponding to *msmK* was increased over two-fold in day 7 biofilms. MsmK acts as a common ATPase for multiple carbohydrate transporters in pneumococcus and mutation of *msmK* reduced colonization in a mouse model [38]. Similarly, in a screen of pneumococcal biofilm mutants, several PTS and ABC transporters were shown to be defective in colonization [42].

Pneumococcal biofilm development also resulted in a quantitative increase in several proteins associated with a transparent phenotype, which plays an important role in colonization and adherence [5], [43]. Tagatose-1, 6-diphosphate aldolase, ABC transporter sugar-binding protein, ADI and ornithine carbamoyltransferase were similarly upregulated in a transcriptional profiling study of *S. pneumoniae* comparing transparent and opaque colony variants [43] and have been linked to pneumococcal colonisation of the airway in several studies [9], [16], [44].

Pneumococcal virulence proteins associated with infection, persistence and competitive fitness were mostly downregulated during biofilm development. NADH oxidase (*nox*) decreased over two-fold in nascent biofilms. NADH oxidase regulates competence, virulence, and pneumococcal persistence through its actions as an oxygen sensor, in detoxifying oxygen, and in improving the efficiency of glucose catabolism [45] and plays an important role in pneumococcal infection in animal models of both pneumonia and otitis media [46]. However, pyruvate oxidase (*spxB*) was significantly upregulated in day 7 biofilms. SpxB appears to be important in carbohydrate selection and capsule production [47], and in reducing competition by other organisms, since pneumococcal extracellular H_2_O_2_ inhibits the growth of competing bacteria [48], [49]. Recently pyruvate oxidase was shown to be essential for pneumococcal aggregate formation and biofilm growth [27] and mutation of *spxB* resulted in changes in colonization and transparent/opaque phenotype expression [47], [50]. Pneumococcal biofilm growth resulted in increased levels of bacteriocin transport accessory protein (Bta). Since small antimicrobial peptides play a role in pneumococcal competition with closely related bacterial species [51] biofilm growth may enhance *S. pneumoniae* fitness [1].

Finally quantitative proteomic analyses of pneumococcal biofilms may be useful in identifying cross-protective protein antigen candidates that target colonization across serotypes. The iTRAQ dataset highlighted both proteins that were modulated between planktonic and biofilm modes of growth, and those stably expressed in both conditions and may be useful in screening potential antigens for mucosal or transcutaneously administered vaccines [52], [53]. Several prominent proteins quantified in iTRAQ proteomic profiles appear to be immunogenic. For example, nearly two-thirds of the 17 immunogenic proteins identified by MALDI-TOF analysis are present in iTRAQ biofilm profiles, including 2 of 2 proteins that were highly immunogenic across all age groups: pyruvate oxidase and α-enolase [54]. Fba and GapDH were other strongly immunogenic glycolytic enzymes and 6PGD, a surface-associated protein with putative adhesin-like activity, was significantly increased in day 7 pneumococcal biofilms. Present in multiple pneumococcal strains and highly immunogenic in adults, 6PGD was also immunoprotective in a mouse model of infection [55]. Although NADH oxidase (nox) was recently evaluated as a potential vaccine candidate that resulted in decreased pneumococcal colonisation in the nasopharynx and lungs in mice [56] iTRAQ data suggest this protein was downregulated in nascent biofilms. The maltose/maltodextrin ABC transporter binding protein MalX, however, also identified as a vaccine candidate, was quantitatively unchanged in nascent biofilms and increased in day 7 biofilm pneumococci [40]. Notably, this protein has also been identified through proteomic analysis to be a widely conserved pneumococcal antigen capable of eliciting mucosal T_H_17 responses that abrogated colonization [53].

*S. pneumoniae* metabolic regulation appears to be of key importance in biofilm growth. Although marked downregulation of glycolysis, was observed, alternative metabolic pathways likely enhance the ability of pneumococcus to adapt to biofilm growth and facilitate survival. Such metabolic proteins may provide novel cross protective target antigens underlying pneumococcal persistence in multiple serotypes.
